# Intraoperative tumor lysis syndrome in a giant teratoma: a case report

**DOI:** 10.1186/s12893-019-0526-4

**Published:** 2019-06-14

**Authors:** Daniel Pindak, Katarina Rejlekova, Miroslav Tomas, Ramadan Aziri, Eva Rovenska, Judita Puskacova, Michal Mego

**Affiliations:** 1 Department of Surgical Oncology, National Cancer Institute Bratislava, Bratislava, Slovakia; 2 Department of Surgical Oncology, Faculty of Medicine, Slovak Medical University, Bratislava, Slovakia; 3 2nd Department of Oncology Faculty of Medicine, Comenius University, Bratislava, Slovakia; 4 Department of Oncology, National Cancer Institute Bratislava, Klenova 1, 833 10 Bratislava, Slovakia; 5 Department of Pediatric Hematology and Oncology, Comenius University Children’s Hospital, Bratislava, Slovakia

**Keywords:** Surgery, Teratoma, Genitourinary neoplasm, Necrosis, Intraoperative

## Abstract

**Background:**

Tumor lysis syndrome is an unusual metabolic emergency in solid tumors. Perioperative occurrence of this syndrome is extremely rare but may have fatal consequences if not detected and treated on time.

**Case report:**

We report a 19-year patient with testicular germ cell tumor after first line chemotherapy with giant growing teratoma syndrome in retroperitoneum. He underwent radical resection, however, perioperatively, a fatal case of heart failure due to unrecognized intraoperative tumor lysis syndrome developed.

**Conclusion:**

Surgeons, anesthesiologists and oncologists should be aware of this complication in order to be prepared for such an emergency.

## Background

Tumor lysis syndrome (TLS) is an oncometabolic emergency resulting from rapid cell death. It is reported that TLS can usually occur as a consequence of tumor-targeted therapy (chemotherapy, embolization therapy and radiation therapy) or spontaneously [1, 2]. Perioperative occurrence of this syndrome is extremely rare but may have fatal consequences if not detected and treated on time [3, 4].

We present a case of a young man who developed a hyperkalemic cardiac failure due to TLS during surgery for a giant retroperitoneal teratoma. The aim of this report is to have the knowledge about the possibility and occurrence of this unusual complication and to be aware of such an emergency.

## Case presentation

### Patient’s history

**Fig. 1:**
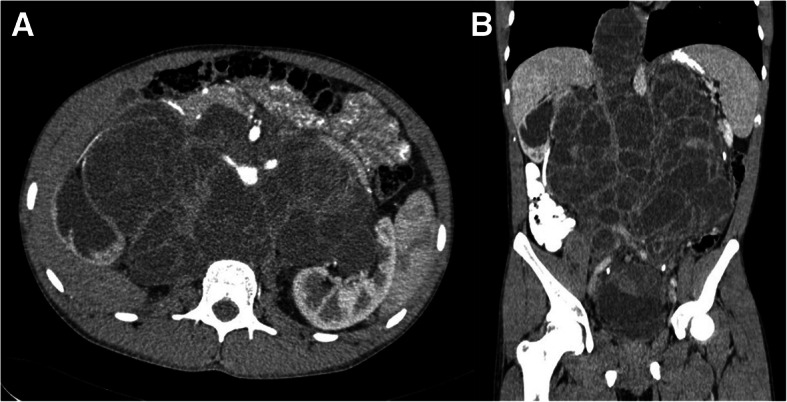
CT scans confirming giant retroperitoneal tumor extending to the posterior mediastinum. **a** – axial, **b** – coronal

A 17-year old boy without significant past medical history presented himself with a 6-month history of back pain in May 2015. Physical examination revealed a huge abdominal mass, and subsequent magnetic resonance imaging verified a giant multifocal tumor with solid and cystic formations filling the space of the retroperitoneum, continuing to the posterior mediastinum and the small pelvis, in transversal diameter measuring 214 × 144 mm. He hadn’t noticed an enlarging mass in the right testicle for several months prior. Consequently, right orchiectomy was performed, and histology revealed mature teratoma. A staging computer tomography (CT) scan confirmed a huge retroperitoneal tumor and revealed also left supraclavicular lymphadenopathy and numerous bilateral lung metastases (Fig. 1). Metastatic involvement and high levels of human beta-choriogonadotropin (bHCG) 23,594 IU/L and alpha-fetoprotein (AFP) 2159 mIU/L classified the patient into the intermediate prognostic group based on the International Germ Cell Cancer Collaborative Group classification [5]. He was treated with 5 cycles of Cyclo-BEP (cyclofosfamide, bleomycin, etoposide, cisplatin) in the Children’s Oncology Hospital with minimal tumor regression and a slow decrease of tumor markers. In that time our institution was consulted, and our head surgeon suggested a few step surgery. However, the patient declined. He was given second line chemotherapy TIP (paclitaxel, ifosfamide and cisplatin), but after one cycle patient decided not to continue. From December 2016 he was followed for 10 months. During this time the disease was stable, there was an almost complete normalization of bHCG and a slightly elevated AFP (21.4 mIU/L) with discrete growing of abdominal tumor mass. Therefore, a diagnosis of growing teratoma syndrome was established. In September 2017, due to clinical deterioration, weight loss, necessity of opioid analgesis to control backache and recurrent acute renal failure after insertion of bilateral nephrostomies, he finally accepted operation.

Patient was admitted 1 week before scheduled surgery for nutritional support, preoperative anesthesiologic evaluation, and isotope renography to evaluate actual renal functions.

### Preoperative surgery planning

Complete tumor resection in the abdomen as well as in the mediastinum was planned, leaving the supraclavicular tumor for subsequent surgery if needed. As isotope renography revealed a functionless right kidney, autotransplantation of the left kidney was planned at the end of the procedure. Patient and family were informed about the probability of losing both kidneys. Together with vascular surgeons we considered performing a two-stage procedure – an extra anatomic bypass (axillo –bifemoral) first, followed by subsequent tumor resection to achieve ischemia-reperfusion time shorter. Based on our previous experiences we decided to perform a one stage procedure while selectively considering the need of the extra anatomic bypass intraoperatively – in regards to the estimated length of the aortal and caval clamping. We planned to perform Y bypass reconstruction of the aorta as well as the inferior caval vein with optional left kidney autotransplantation according to intraoperative finding on the left renal vessels.

### Procedure

**Fig. 2:**
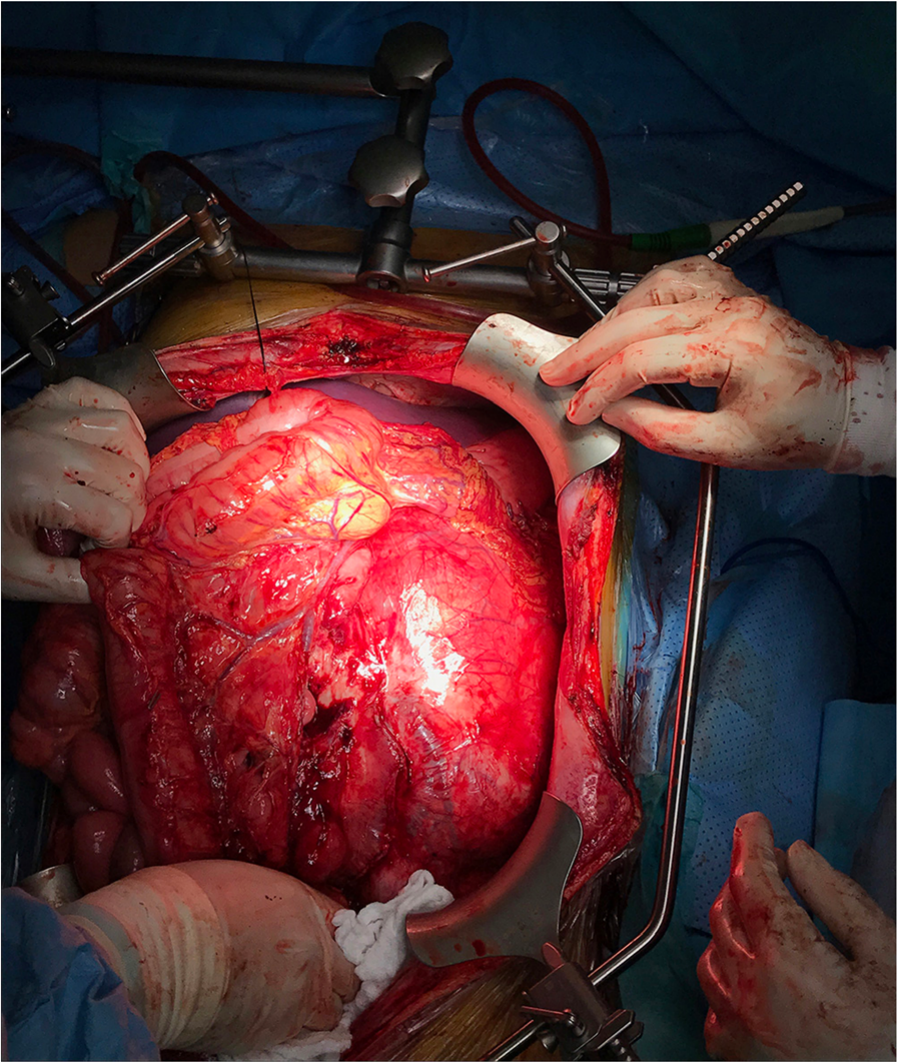
Intraabdominal view after laparotomy – abdominal cavity completely filled by the tumor

**Fig. 3:**
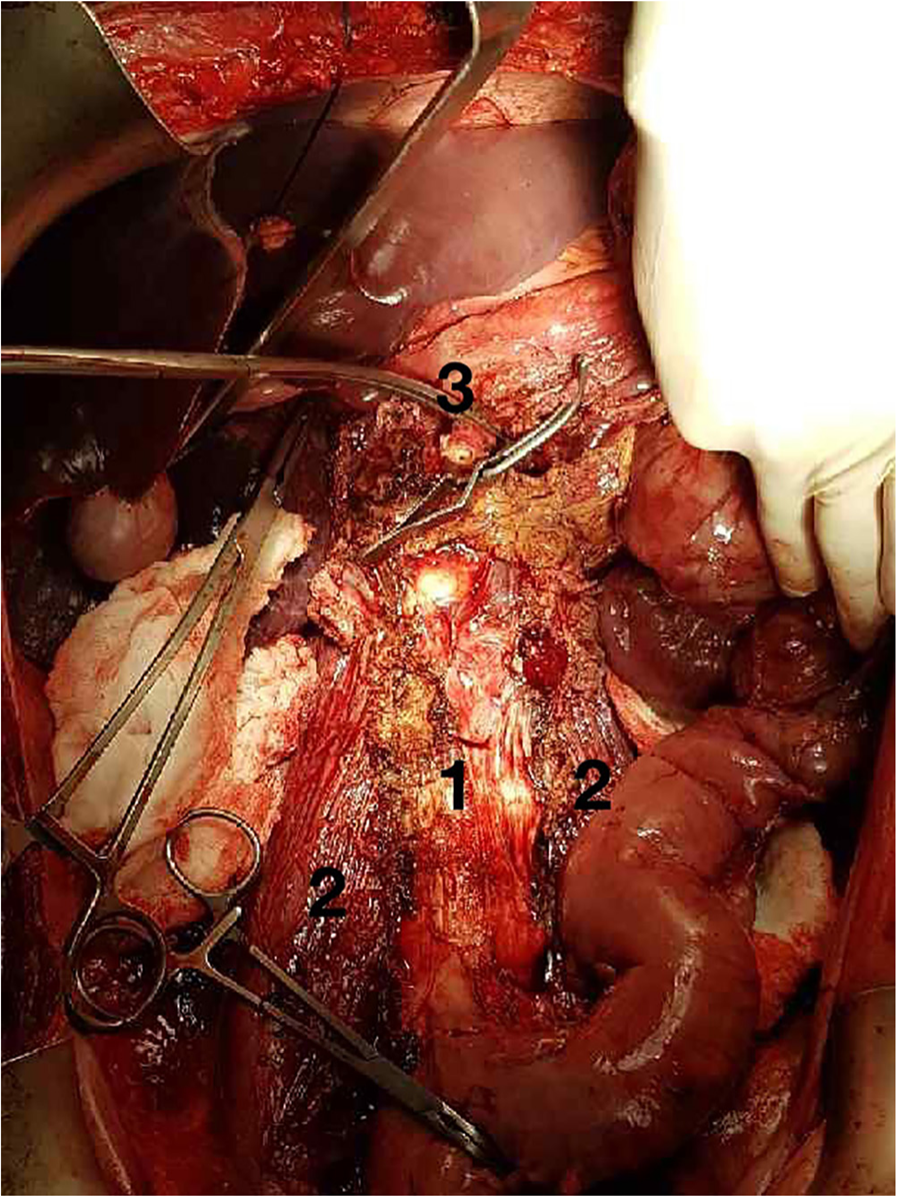
Intrabdominal view after tumor resection (1 vertebral column, 2 psoas muscles, 3 interrupted aorta)

After the extensive laparotomy allowing good access, a huge tumor completely filling the abdominal cavity was visible (Fig. 2). We found the tumor resectable as it was possible to access the aorta safely just in the level of superior mesenteric artery (SMA). Due to encasing of the left kidney’s vessels by the tumor mass, autotransplantation was not possible. We started with tumor resection, a complete mobilization of liver, small bowel and large bowel was performed yet no infiltration of above-mentioned structures was presented. During mobilization of the duodenum and pancreatic head and body we found infiltration of the third part of the duodenum so segmental resection using stapling devices was performed. Afterwards we encircled the aorta above the renal arteries and below the SMA, and the inferior caval vein (IVC) below the caudate lobe, common iliac arteries and external iliac veins. After medial frenotomy we did mobilization of the mediastinal part of the tumor. As we had very good exposure and complete access to important vessels, we estimated that the time of ischemia-reperfusion will be certainly less than 120 min, so we decided to not use extraanatomic bypass. After administration of Heparin – in the dose of 100 units per kilogram, we put clamps on the aorta, IVC and iliac vessels and we finished the resection of the tumor by separating it from the vertebral columns and psoas muscles (Fig. 3). The function of the left kidney was preserved until clamping during the whole resectional part of the procedure, with intraoperative urine output 0.66 ml/kg/hour in average. Subsequently we started reconstruction: we did proximal aortal anastomosis and due to the short segment of the aorta below the celiac trunk, we did reinsertion of the SMA to the prosthesis. During the creating of the distal arterial anastomosis the anesthesiologist reported cardiac arrhythmias with no pulse waves detected. Complete resuscitation using manual direct heart massage and complete pharmacologic support was started. Emergent perioperative blood tests revealed severe hyperkalemia (8.3 mmol/l), severe acidosis (pH 7.05), hyperphosfatemia (2.98 mmol/l) and hypercalcaemia (2.87 mmol/l) due to the administration of calcium chloratum after each blood transfusion - 3 units in total. The serum creatinine level was slightly elevated (135 μmol/l) and serum hemoglobin after substitution was 98 g/l. Even after complete cardiopulmonic resuscitation, administration of natrium bicarbonate, i.v. glucose with insulin, another calcium chloratum with the aim to decrease serum potassium level and after serial electric defibrillations due to ventricular fibrillation, the patient developed asystolia and, after 40 min of complete resuscitation, exitus was stated. After reviewing the clinical case and the evidence, cardiac failure was established as the cause of patient’s death by the pathologist after autopsy.

**Fig. 4:**
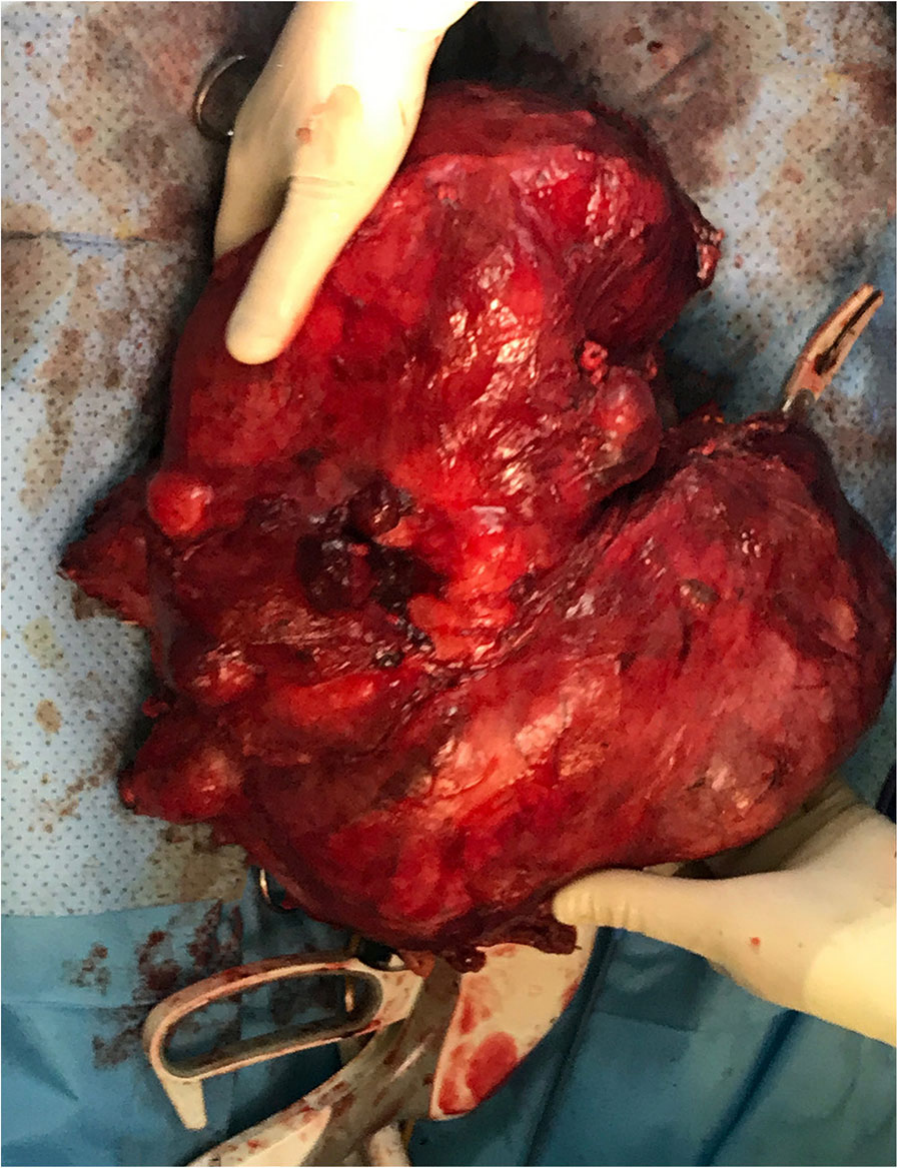
Resected specimen - large encapsulated tumor mass measuring 33x25x13 cm

**Fig. 5:**
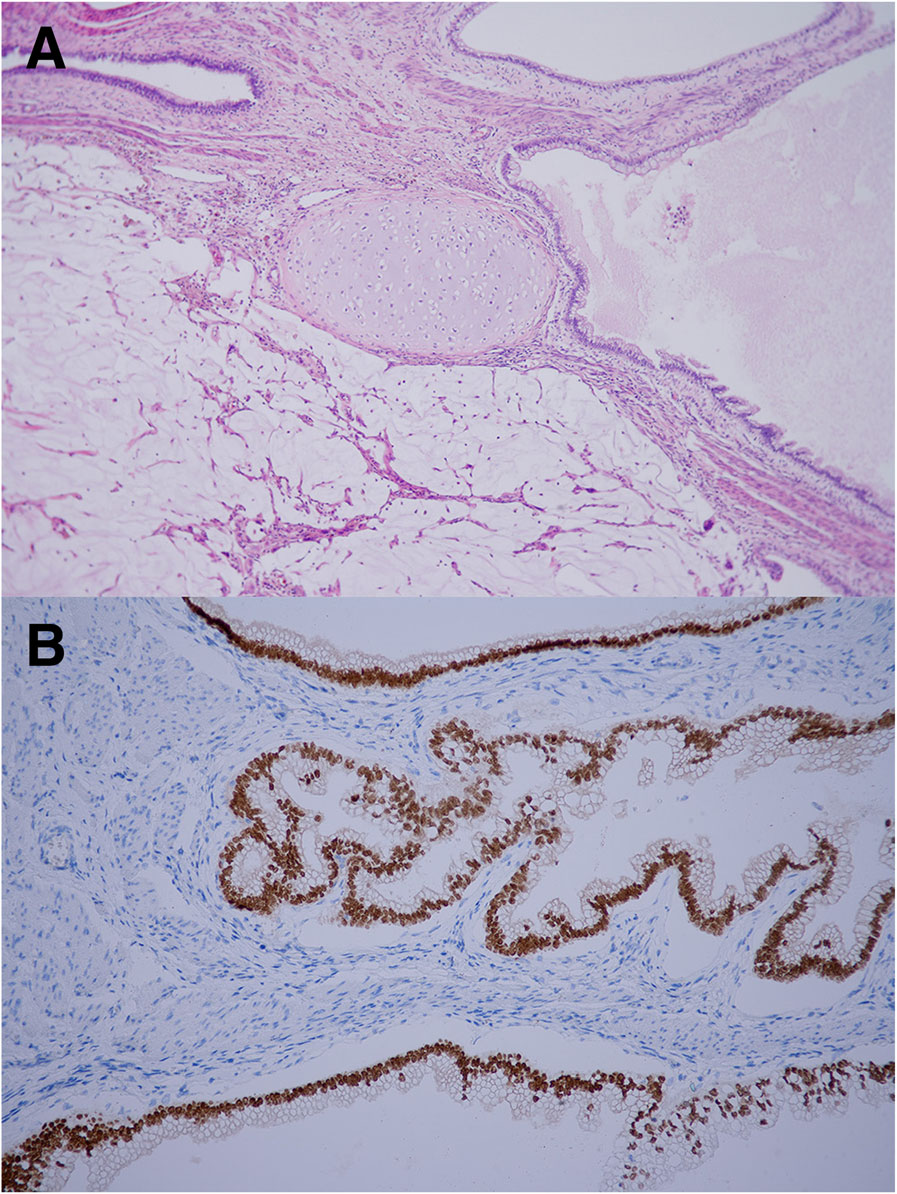
Histology. **a** - organoid morphology of mature teratoma: cysts lines by respiratory type of epithelium, cartilage and mucoid areas, **b** - strong nuclear staining of CDX2 – marker to detect epithelium with gastrointestinal differentiation

The final pathology report describes large encapsulated tumor mass mesauring 33x25x13 cm, the mass show grey-brown color, with multifocal cysts with gelatinous and serous material (Fig. 4). Histology showed tissue arrangements mimicking organoid morphology, mature glandular tissue of the skin, respiratory tract and gastrointestinal tract: cysts lines by respiratory type of epithelium and intestinal epithelium with goblet cells, surrounded by smooth muscle and adipose tissue and cartilage, intraluminally was found mucoid areas with multinucleated giant cells, without cytologic atypia or necrosis and minimal mitoses, without original structures of lymphoid tissue (Fig. 5). It was not provided 12p amplification. Immunohistochemistry: AE+/3+, CD117-, CEA+, PLAP-, HCG-, CD30-, LCA-, CDX2+. The final diagnosis was postchemoterapy lymph node metastases from mature teratoma.

## Discussion and conclusions

TLS is basically characterized by hyperkalemia, hyperphosphatemia and hyperuricemia, which has been described by Cairo and Bishop in their definition for both laboratory and clinical criteria [6, 7]. TLS is a relatively rare event in patients with solid tumors and reports about its presentation in testicular cancer are even less common, counting no more than 10 cases in the review articles published up to now [1, 8, 9]. Most often TLS occurred in patients with solid cancers as a result of some form of cancer-targeted therapy (radiation, chemotherapy or hormonal therapy) and even after biopsies [1]. Reports about its intraoperative presentation are even less common, including only a few case reports and no TLS in patients with growing teratoma syndrome was reported [3, 8].

We arrived at the provisional diagnosis of TLS after taking into account a very unusual cause of death with no other explanation. Patient was stabilized for almost the whole course of the surgery with very fast deterioration during the final part of the surgery, with no signs of hypovolemia and before planned reperfusion was done. We assumed that partial tumor devascularisation during its mobilization and tumor manipulations and serial compressions during resection led to multifocal partial tumor necrosis with subsequent release of potassium and other products from the ischemic tumor with subsequent severe metabolic disturbances finally leading to patient’s death.

Clinicians should keep in mind that patients with solid tumors may develop this potentially deadly syndrome. Based on the current literature it seems that patients with advanced and metastatic tumors may be at risk for TLS. Other potential risk factors might be the presence of elevated baseline creatinine and decreased renal function, elevated lactate dehydrogenase, elevated phosphorus, elevated potassium and elevated uric acid [8, 10]. It is also essential to keep in mind that TLS can occur intraoperatively in giant solid cancers. Our patient had an advanced, metastatic tumor, as well as decreased renal function, and therefore was at high risk for developing TLS.

From a surgical point of view, after experiencing this complication, we suggest and will perform stricter perioperative potassium level monitoring in the future, as hyperkalemia is the most life-threating manifestation of TLS and was also the main manifestation in the presented case. We also suggest clamping outflow from the tumor as soon as possible during the surgery, even if it will require the use of extracorporeal circulation support or two stage procedure with extracorporeal bypasses done during the first stage. We will also consider intraoperative use of renal replacement therapy in those patients with planned giant abdominal tumor resections with preoperative renal dysfunction. These kinds of procedures should be performed in centers where such intraoperative interventions are available.

For the first time we report a case of tumor lysis syndrome in giant retroperitoneal teratoma, triggered by surgery. The aim of our paper is to draw attention to this unusual intraoperative complication and make surgeons, anesthesiologists and oncologists aware of this complication and its occurrence even in solid well-differentiated tumors.

## Data Availability

Authors can confirm that all relevant data are included in the article.

## Abbreviations

AFP: Alpha-fetoprotein
bHCG: Beta-choriogonadotropin
CT: Computer tomography
Cyclo-BEP: Cyclofosfamide, bleomycin, etoposide, cisplatin, TIP paclitaxel, ifosfamide and cisplatin
IVC: Inferior caval vein
SMA: Superior mesenteric artery
TLS: Tumor lysis syndrome
